# Virome of *Ixodes ricinus*, *Dermacentor reticulatus*, and *Haemaphysalis concinna* Ticks from Croatia

**DOI:** 10.3390/v14050929

**Published:** 2022-04-29

**Authors:** Stephen Sameroff, Rafal Tokarz, Marko Vucelja, Komal Jain, Alexandra Oleynik, Marko Boljfetić, Linda Bjedov, Rachel A. Yates, Josip Margaletić, Christopher A. L. Oura, Walter Ian Lipkin, Lidija Cvetko Krajinović, Alemka Markotić

**Affiliations:** 1 Center for Infection and Immunity, Mailman School of Public Health, Columbia University, New York, NY 10032, USA; scs2178@cumc.columbia.edu (S.S.); rt2249@cumc.columbia.edu (R.T.); kj2230@cumc.columbia.edu (K.J.); axx@publichealth.columbia.edu (A.O.); rachelay@gmail.com (R.A.Y.); wil2001@cumc.columbia.edu (W.I.L.); 2 School of Veterinary Medicine, The University of the West Indies, St. Augustine, Trinidad and Tobago; christopher.oura@sta.uwi.edu; 3 Department of Epidemiology, Mailman School of Public Health, Columbia University, New York, NY 10032, USA; 4 Department of Forest Protection and Wildlife Management, Faculty of Forestry and Wood Technology, University of Zagreb, 10000 Zagreb, Croatia; marko.vucelja@sumfak.unizg.hr (M.V.); mboljfetic@oikon.hr (M.B.); linda.bjedov@sumfak.unizg.hr (L.B.); josip.margaletic@sumfak.unizg.hr (J.M.); 5 Research Department, University Hospital for Infectious Diseases, 10000 Zagreb, Croatia; lcvetko@bfm.hr; 6 Faculty of Medicine, University of Rijeka, 51000 Rijeka, Croatia; 7 School of Medicine, Catholic University of Croatia, 10000 Zagreb, Croatia

**Keywords:** tick-borne diseases, high throughput sequencing, Croatia, *Flaviviridae*, *Rhabdoviridae*, *Nyamiviridae*, *Bunyavirales*

## Abstract

Tick-borne diseases are a serious threat to both public and veterinary health. In this study, we used high-throughput sequencing to characterize the virome of three tick species implicated in the spread of vector-borne disease throughout Croatia. Ten viruses were identified, including seven potential novel species within the viral families *Flaviviridae*, *Nyamiviridae*, *Rhabdoviridae*, *Peribunyaviridae*, *Phenuiviridae*, and *Nairoviridae*.

## 1. Introduction

The incidence of vector-borne diseases in Europe is increasing. This is likely due to expanding vector ranges linked to climate change, increased human exposure due to incursions into wildlife habitats, and improved case ascertainment due to enhanced testing for vector-borne diseases [1,2,3,4,5,6]. Tick-borne diseases (TBDs) are responsible for the highest disease burden of vector-borne diseases within Europe [2]. Throughout Europe, the emphasis for TBD has primarily focused on Lyme borreliosis (LB) and tick-borne encephalitis (TBE). Incidence of TBE remains consistent across Europe at approximately 0.6 cases per every 100,000 people [7], aided in part by vaccination programs [8]. Conversely, LB cases are increasing, with an annual conservative estimate of approximately 85,000 cases on the continent [2]. Other tick-borne pathogens commonly found in the region include Crimean–Congo hemorrhagic fever virus (CCHFV), *Anaplasma phagocytophilum*, *Babesia divergens* and *Rickettsia conorii* [1,3,6,9,10]. While there have been improvements in surveillance programs and testing, studies focused on the characterization of the tick microbiome are scant, partly due to the expense of high-throughput sequencing (HTS) [11].

Croatia has carried out active surveillance for ticks and TBDs over the past thirty years, resulting in a total of 22 tick species spanning five genera documented in the country [12,13,14,15]. The primary focus has been on surveillance for tick-borne encephalitis virus (TBEV) and *Borrelia burgdorferi* sensu lato, along with the identification of ticks and reservoir hosts involved in the transmission cycles of these agents [16,17,18,19,20,21,22,23]. Additionally, a survey among rodents in the northern region of Croatia identified a tick-borne pathogen of concern, *Borrelia miyamotoi*, in approximately 4% of the rodent population [24]. In other recent studies, researchers have investigated the prevalence of specific agents of disease, including *Babesia* spp., *Anaplasma* spp., and *Ehrlichia* spp. [25,26,27]. Some agents endemic to Croatia have not been included in surveillance programs, such as Bhanja virus [28,29], a bunyavirus that is pathogenic in both humans and ruminants.

Over the last decade, several novel tick-borne viruses have emerged globally as agents of human disease, including Dabie bandavirus, also known as severe fever with thrombocytopenia syndrome virus (SFTSV) [30], Heartland virus [31], Bourbon virus [32], and most recently the Jingmen tick virus [33]. The emergence of these pathogens has highlighted the need for a greater understanding of the tick virome. This study utilized unbiased high throughput sequencing with an aim to more closely explore the virome of ticks endemic to Croatia.

## 2. Materials and Methods

### 2.1. Collection

**Figure 1:**
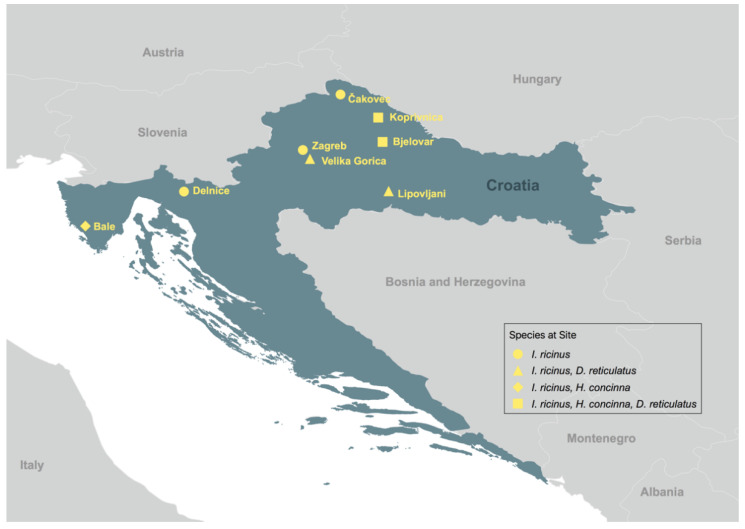
Tick collection sites and the species identified at each site. This map was generated using QGIS 3.4.2 using DIVA GIS shape files.

Ticks were collected by dragging method [34,35,36] at eight different locations throughout Croatia (Figure 1) between 2012 and 2017 during spring and fall seasons.

Dragging took place using a 1 m × 1 m white flannel cloth for one hour each with 15 min per transect per site visit. After collection, the samples were sorted by species (using identification keys), collection date, and location. Identification of ticks was conducted using the standard key for European ticks [37] via stereomicroscope. Ticks were stored in 100% ethanol at −80 °C.

### 2.2. Extraction and Species Identification

Prior to nucleic acid extraction, ticks (separated by species, location, and date) were each washed in 1 mL of hydrogen peroxide followed by three washes with 1 mL of ultraviolet-irradiated, nuclease-free water and then air-dried. Individual ticks were then transferred into a 1.7 mL microcentrifuge tube containing 100 µL of viral transport media (VTM) (Becton Dickinson, Franklin Lakes, NJ, USA) and homogenized. Total nucleic acid (TNA) was extracted from 33 µL of tick homogenate on the EasyMag platform (BioMerieux, Marcy-l’Étoile, France) [38] and eluted in 40 µL of elution buffer. From each sample, 11 µL of the TNA was aliquoted for RT-PCR while the remainder was stored at −80 °C.

To confirm the tick species sorted by identification keys, a barcoding PCR was performed using primers targeting the 16 s rRNA mitochondrial gene. Using 1 µL of cDNA as a template, the cycling conditions were as follows, 95 °C for 10 min, followed by 35 cycles of 95 °C for 30 s, 55 °C for 40 s, and 72 °C for 40 s, and a final step of 72 °C for 5 min. All PCR products were confirmed using Sanger sequencing.

### 2.3. Sequencing and Bioinformatics

Following species confirmation, 33 µL of original VTM homogenate from individual ticks were pooled according to species (*n* = 20 per pool for *D. reticulatus*, *n* = 33 for *I. ricinus*, and *n* = 22 for *H. concinna*) to create libraries for HTS. Before extraction on the EasyMag platform (BioMerieux, Marcy-l’Étoile, France), 300 µL of pooled material was purified to enrich for viral particles. Pools were filtered (0.45 µM) then treated with RNase A for 15 min at room temperature and Turbo DNase and Benzonase (MilliporeSigma, Burlington, MA, USA) for 30 min at room temperature. This method degrades nucleic acids that are not protected by the presence of a viral capsid. TNA (11 µL) from each tick pool was subjected to first and second-strand cDNA synthesis with Super Script IV reverse transcriptase (Invitrogen, Waltham, MA, USA) and exo-Klenow fragment (New England Biolabs, Ipswich, MA, USA), respectively. Double-stranded DNA was processed for the library construction using a KapaHyperPlus kit (Roche, Basel, Switzerland). Sequencing was performed on the Illumina NextSeq 550 system (Illumina, San Diego, CA). The demultiplexed FastQ files were adapter trimmed using the Cutadapt program (v3.0) [39]. Adapter trimming was followed by generation of quality reports using FastQC software (v0.11.5) [40], which were used to determine filtering criteria based on the average quality scores of the reads, read length, homopolymeric reads, nucleotide bias and quality scores at the ends of the reads. The reads were quality filtered and end trimmed with PRINSEQ software (v0.20.3) [41]. Host background levels were determined by mapping filtered reads against a tick reference database (consisting of all *Ixodes scapularis*, *Amblyomma americanum*, and *Dermacentor variabilis* sequences present in GenBank as of August 2019) using Bowtie2 mapper (v2.2.9) [42]. The host-subtracted reads were de novo assembled using the MIRA (4.0) and MEGAHIT (1.2.8) assemblers [43,44]. Contigs and unique singletons were subjected to homology search using MegaBLAST against the GenBank nucleotide database. Sequences that showed low or no homology at the nucleotide level were subjected to a BLASTX homology search against the viral GenBank protein database.

### 2.4. Phylogenetic Analysis

Protein sequences were aligned using ClustalW in Geneious 10.2.4. Alignments were filtered using Gblocks [45] to remove poorly aligned regions and gaps within the alignments. Phylogenetic trees were constructed with MEGAX 10.1.7 [46]. The robustness of each node was determined using 1000 bootstrap replicates using a maximum likelihood (ML) method employing an LG+G+I model with nearest-neighbor interchange (NNI) determined to be the best model through a ML fit of 56 different amino acid substitution models. Trees were populated with RefSeq sequences from all ICTV recognized species in addition to the closely related tick-borne virus with similarity to the viruses identified in this study that has yet to be recognized and classified by ICTV.

## 3. Results

One hundred and seventy-five ticks (*D. reticulatus* = 120, *I. ricinus* = 33, and *H. concinna* = 22) were selected as a representative set of the total samples collected throughout Croatia from 2012–2017. These individual ticks were combined into eight pools (6 = *D. reticulatus* [*n* = 20/pool], 1 = *I. ricinus* and 1 = *H. concinna*) and were sequenced using an Illumina NextSeq, generating a total of 278,018,480 raw reads. After quality filtration and host subtraction, 61,245,248 reads remained which were assembled into 768,789 contigs, with 951 identified as viral in origin through BLASTN and BLASTX (additional bioinformatic information for the individual pools in Supplemental Table S1). Sequences of ten putative viruses were identified, with seven representing novel species. Seven viruses were identified within the *I. ricinus* pool, with the remaining three identified within the *D. reticulatus* pools (Table 1). No viral sequences were identified in the *H. concinna* pool.

### 3.1. Flaviviridae

**Figure 2:**
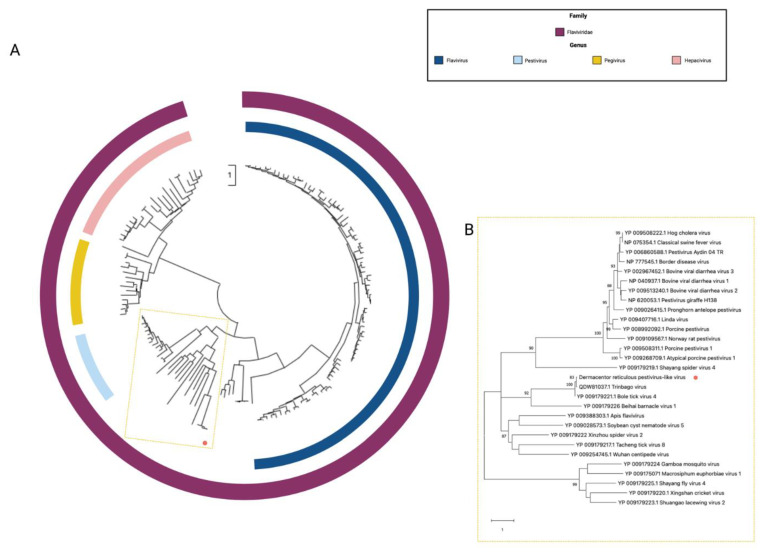
Phylogenetic relationship of family *Flaviviridae* based on an alignment of a 682 aa fragment of the NS5: (**A**) Alignment of all species belonging to family *Flaviviridae*; (**B**) enhanced region showing the relationship of the unclassified pestivirus-like group in relation to pestivirus.

Sequences for a novel viral species belonging to family *Flaviviridae*, tentatively named *Dermacentor reticulatus* pestivirus-like virus 1, were identified in five of the *D. reticulatus* pools. *Dermacentor reticulatus* pestivirus-like virus 1 (DRPV1) comprises a single polyprotein that shares closest homology within the NS3 and NS5 (<30% aa identity) of viruses within the genus *Pestivirus*. Phylogenetic analysis (Figure 2) clusters DRPV1 with other recently identified pestivirus-like viruses, Bole tick virus 4 [47] and Trinbago virus [48].

### 3.2. Nyamiviridae

**Figure 3:**
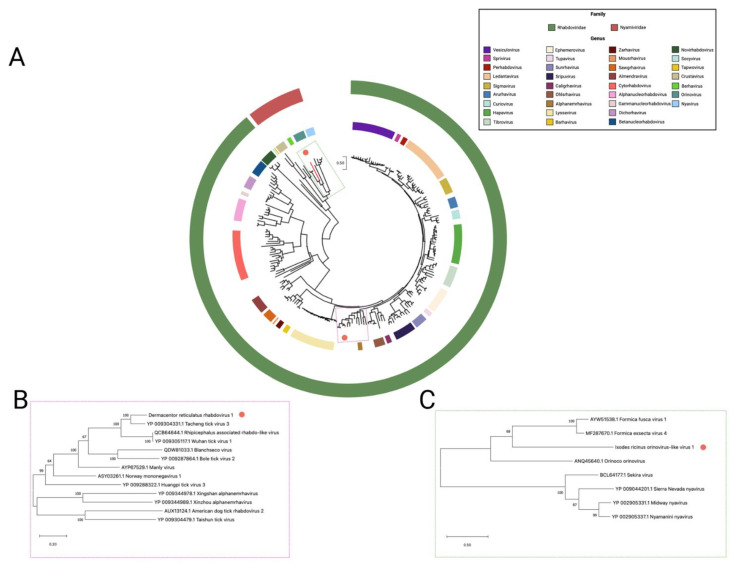
Phylogenetic relationship of families *Nyamiviridae* and *Rhabdoviridae* based on an alignment of a 532 aa fragment of the RNA-dependent RNA polymerase: (**A**) Represents all species belonging to families *Nyamiviridae* and *Rhabdoviridae*; (**B**) enhanced region showing the relationship of unclassified tick-borne rhabdoviruses and the next closest genus *Alphanemrhabdovirus*; (**C**) enhanced region showing the relationship of orinovirus and nyavirus.

A sequence with similarity to viruses within genus *Orinovirus* was identified within the *I. ricinus* pool. Tentatively called *Ixodes ricinus* orinovirus-like virus 1, this highly divergent virus is only 42% aa similar within the polymerase to the next closest relative, Hymenopteran orino-related virus [49]. *Ixodes ricinus* orinovirus-like virus 1 (IROV1) represents the first virus from the family *Nyamiviridae* identified from a hard tick species. Its genome consists of six putative ORFs, although only three, the nucleoprotein, the glycoprotein, and the polymerase, could be identified through homology searches. Phylogenetic analysis of the polymerase gene (Figure 3) clusters IROV1 with other recently identified orinoviruses, formica fusca virus 1 and formica exsecta virus 4.

### 3.3. Rhabdoviridae

A novel rhabdovirus species, tentatively named *Dermacentor reticulatus* rhabdovirus 1, was identified in five out of the six *D. reticulatus* pools. We identified four ORFS within *Dermacentor reticulatus* rhabdovirus 1 (DDR1), with three displaying homology to conserved domains associated with the family *Rhabdoviridae*; the nucleocapsid, the matrix protein, and an RNA-dependent RNA polymerase; however, the precise number of ORFS is unknown. The fourth ORF does not share any known homology with any known ORFs. DRR1 clusters with Tacheng tick virus 3 (Figure 3) [50], which was identified in *Dermacentor* *marginatus* ticks.

### 3.4. Nairoviridae

**Figure 4:**
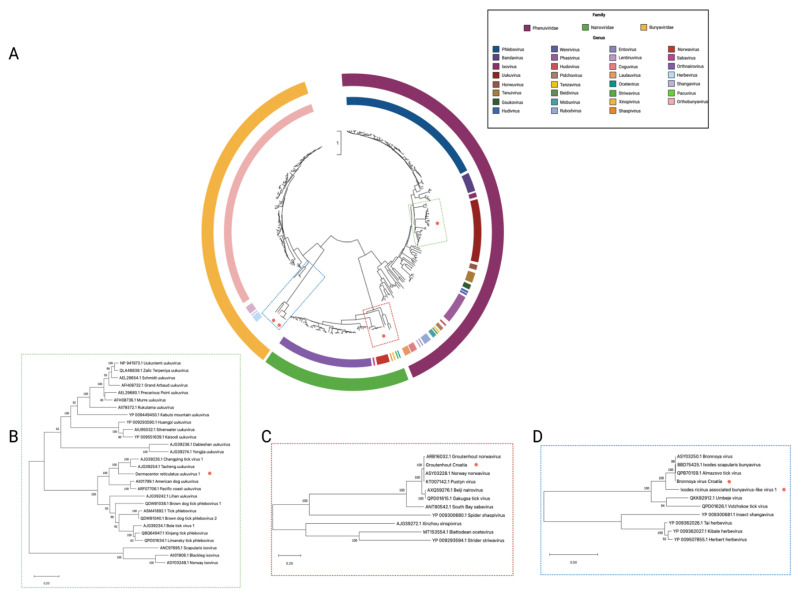
Phylogenetic relationship of families *Phenuiviridae*, *Nairoviridae*, and *Bunyaviridae* based on an alignment of a 368 aa fragment of the RNA-dependent RNA polymerase: (**A**) Represents all species belonging to families *Phenuiviridae*, *Nairoviridae*, and *Bunyaviridae*; (**B**) enhanced region showing the relationship of uukuvirus and ixovirus; (**C**) enhanced region showing the relationship of norwavirus, sabavirus, shaspivirus xinspivirus, octevirus, and striwavirus; (**D**) enhanced region showing the relationship of unclassified tick-borne bunyavirus-like viruses and herbevirus.

Partial sequences of a nairovirus-like viral genome were identified within the *I. ricinus* pool, consisting of an L-segment fragment encoding the RdRp and the complete S-segment encoding the nucleocapsid. Sequences representing an M-segment were not identified. This putative virus shares > 95% aa similarity with the Grotenhout virus, Norway nairovirus 1, and Pustyn virus, all of which lack M-segment sequences [51]. All M-segment deficient nairoviruses to date have been identified in *Ixodes* tick species and cluster with their host tick species (Figure 4), suggesting these viruses may have co-evolved with their tick hosts.

### 3.5. Phenuiviridae

Sequences for a novel uukuvirus, tentatively called *Dermacentor reticulatus* uukuvirus 1, were identified in five out of six *D. reticulatus* pools. *Dermacentor reticulatus* uukuvirus 1 (DRU1) consists of two segments encoding for the RdRp and the nucleoprotein. However, as with many other recently identified tick-borne uukuviruses, DRU1 appears to be deficient of an M-segment encoding the glycoprotein. Phylogenetic analysis (Figure 4) shows that this virus forms a monophyletic clade with other M-segment deficient uukuviruses isolated from other *Dermacentor* species worldwide.

### 3.6. Peribunyaviridae

Two bunyavirus-like viruses were identified within the lone *I. ricinus* pool. The first was highly similar at the aa level (>98% in the polymerase and >85% in the glycoprotein) to Bronnoya virus (Figure 4), a bunyavirus-like virus identified within *I. ricinus* ticks in Norway [51]. The second, tentatively called *Ixodes ricinus* bunyavirus-like virus 1, shared a similar genome structure with Bronnoya virus but was much more divergent with only <55% aa similarity within the polymerase (Figure 4) and <47% aa similarity in the glycoprotein. We could only identify two out of the three segments that are traditionally part of the bunyavirus genome, the L and the M.

### 3.7. Unclassified Viral Sequences

Three sequences of putative arthropod associated viruses were identified within *I. ricinus*: *Ixodes ricinus* picorna-like virus 1 (IRPV1), *Ixodes ricinus* sobemo-like virus 1 (IRSV1), and *Ixodes ricinus* noda-like virus 1 (IRNV1). We obtained what we assume to be complete uninterrupted coding segments for these highly divergent viruses, based on their length compared to their closest genetic relatives. These viral sequences provided few comparative hits within the protein domain database, and even when a hit occurred, they exhibited very low identity. Therefore, no phylogenetic trees were generated for these viral sequences, as they would ultimately not provide accurate phylogenetic relationships.

## 4. Discussion

This study focused on characterizing the virome of questing *D. reticulatus*, *I. ricinus*, and *H. concinna* ticks from Croatia. The outcome from our sampling effort supports the results throughout various European regions, showing that *I. ricinus*, *D. reticulatus*, and *H. concinna* are three of the most abundant questing ticks within the region [4,52,53]. All three tick species have been heavily implicated in the transmission of TBDs throughout the region. *I. ricinus* is the principal vector of the agents of LB along with *A. phagocytophilum*, and the European strain of TBEV [6]. *D. reticulatus* is the primary vector of *Babesia canis*, an important veterinary pathogen found throughout Europe [54], along with the clinically relevant human pathogen Omsk hemorrhagic fever virus [55]. *D. reticulatus* has also been linked with TBEV, two spotted fever group rickettsiae *Rickettsia raultii* and *Rickettsia slovaca*, *Anaplasma marginale*, *Babesia caballi*, and *Theileria equi* [56]. *H. concinna* has been linked with several tick-borne agents, including *Francisella tularensis*, *Coxiella burnetii*, *Rickettsia* spp., *Babesia* spp., *Anaplasma* spp., TBEV and SFTSV/Dabie bandavirus [53].

Despite examining more *D. reticulatus* pools, we identified a greater number of viral sequences in *I. ricinus*. Additionally, no viral sequences were identified within the *H. concinna* pool. Varying levels of viral diversity have been shown in different tick species within Trinidad and Tobago [48]. In a study of *Haemaphysalis* *longicornus* ticks in the US, no viral sequences were identified [57]. Combined, these studies support a hypothesis that tick species can harbor varying levels of viral diversity. Interestingly, no viral sequences for known TBVs endemic to the region, such as TBEV and Bhanja virus, were identified in the sequencing data. This most likely can be attributed to the low prevalence of these viruses within the tick populations along with sampling bias. For example, data show that the prevalence of TBEV maintained within *I. ricinus* populations in Croatia is around 2% [21]. Since we only examined 33 *I. ricinus* ticks, it is unlikely we would identify a positive tick.

One of our most notable findings was the identification of IROV1 within *I. ricinus*. The genus *Orinovirus* is closely related to *Nyavirus*, a genus containing several TBVs identified within soft-tick species and their avian hosts [58,59,60]. Orinoviruses have also been identified in other arthropods [61,62]; however, to our knowledge, IROV1 is the first putative virus within family *Nyamiviridae* to be found within an *Ixodidae* species. There is evidence that these viruses may potentially cause animal disease. Nyaviruses have been isolated from the brain of a dead European starling and caused mortality in experimentally infected newborn mouse pups after intracranial inoculations [63]. Although no human disease has been documented, *I. ricinus* is a frequent ectoparasite of humans.

Three of the viruses identified in this study appear to be missing essential components of their genomes, or the segments are sufficiently different from known segments that they were not identified as viral segments in this study. This is a trend only seen within tick virome studies and, to the best of our knowledge, has not been reported in non-tick metagenomic analyses. IRBV1 and the Croatian strain of Bronnoya virus lack S-segments, which encode the bunyavirus nucleoproteins that facilitate virion assembly, promote virion stability, and support primary transcription [64]. Similarly, DRU1 is deficient in the glycoprotein-encoding M-segment. Phlebovirus glycoproteins are necessary for cellular entry and for vesicle formation within infected cells [65]. There have been conflicting reports on the infectivity of these segment deficient viruses within the vertebrate hosts parasitized by these ticks. A study from Thailand reported no evidence of an adaptive immune response consistent with infection in parasitized humans and animals to any of the segment deficient viruses identified within the ticks [66]. In a study from China, the presence of serum antibodies to an M-segment deficient phlebovirus, YN tick-associated phlebovirus 1, was reported in 4% of cattle from an endemic region [67]. Immunoreactivity was also found in several other viruses recently identified through tick virome analysis that had previously been hypothesized to be tick endosymbionts.

The viral sequences identified in this study highlight the remarkable diversity of tick-borne viruses. The scope of our work was limited to identifying viral sequences within *I. ricinus*, *D. reticulatus*, and *H. concinna* and we can only speculate as to the pathogen potential of these viruses. Future work is required to determine the transmissibility of these viruses, and the potential for causing human or animal disease.

## Figures and Tables

**Table 1:** Summary of viruses identified.

Name	Closest Relative	Family	% Identity aa	Tick Species	Genome Length nt
*Dermacentor reticulatus* pestivirus-like virus 1	Bole tick virus 4	Flaviviridae	86%	*D. reticulatus*	16340
*Dermacentor reticulatus* rhabdovirus 1	Tacheng tick virus 3	Rhabdoviridae	72%	*D. reticulatus*	10313
*Dermacentor reticulatus* phlebovirus-like virus 1	Tacheng tick virus 2	Phenuiviridae	65% 48%	*D. reticulatus*	L-6609 S-1557
*Ixodes ricinus* orinovirus-like virus 1	Formica exsecta virus 4	Nyamiviridae	37%	*I. ricinus*	10148
Bronnoya virus	Bronnoya virus	Peribunyaviridae	98% 84%	*I. ricinus*	L-9121 M-4116
*Ixodes ricinus* associated bunyavirus-like virus 1	Bronnoya virus	Peribunyaviridae	55% 47%	*I. ricinus*	L-9180 M-4287
*Ixodes ricinus* picorna-like virus 1	Hubei picorna-like virus 53	Unclassified	25%	*I. ricinus*	14146
*Ixodes ricinus* sobemo-like virus 1	Hubei sobemo-like virus 47	Unclassified	51%	*I. ricinus*	2669
Groutenhout norwavirus	Groutenhout norwavirus	Orthonairoviridae	Incomplete 98%	*I. ricinus*	L-incomplete S-3707
*Ixodes ricinus* noda-like virus 1	Providence virus	Unclassified	37%	*I. ricinus*	4421

## Data Availability

Sequences for this study are deposited under project code PRJNA802541.

## Supplementary Materials

The following supporting information can be downloaded at: https://www.mdpi.com/article/10.3390/v14050929/s1. Table S1. Table with sequencing read counts per pool and genome coverage and depth for the viruses identified in each pool.
